# Morphological, physiological, and biochemical responses of two industrial hemp (*Cannabis sativa* L.) cultivars to different levels of topping

**DOI:** 10.1186/s42238-026-00410-2

**Published:** 2026-03-06

**Authors:** Spyridoula Chavalina, Vasileios Ioannidis, Dimitrios Bilalis, Fotini Lamari, George Zervoudakis, Georgios Salachas

**Affiliations:** 1 Department of Agriculture, Laboratory of Plant Physiology and Nutrition, University of Patras, New Buildings, Messolonghi, 302 00 Greece; 2 Laboratory of Pharmacognosy & Chemistry of Natural Products, Department of Pharmacy, University of Patras, University Campus of Patras, 265 04, Patras, Greece; 3 Department of Crop Science, Laboratory of Agronomy, Agricultural University of Athens, Iera Odos 75, 118 55, Athens, Greece; 4 Department of Agriculture, Laboratory of Plant Physiology and Biochemistry, University of Patras, New Buildings, Messolonghi, 302 00 Greece

**Keywords:** Apical dominance, Biomass yield, Physiological parameters, Cannabidiol, Cannabigerol

## Abstract

**Background:**

For the cultivation of hemp (*Cannabis sativa* L.), topping (the removal of the apical meristem) is a key cultivation practice, affecting plant morphology, physiological function, biomass allocation, and the yield of inflorescences and cannabinoids. The present study investigated the effect of topping on two monoecious industrial hemp cultivars (‘Enectaliana’ and ‘Santhica 70’).

**Methods:**

The experiment was conducted from April to June 2024 in a greenhouse using 10 L pots, following a completely randomized design with four treatments: no topping and topping above the 4th, 5th, and 6th node from the base of the main stem. During the experimental period, the mean daily air temperature was 26.0 °C and the mean relative humidity was 54.4%. Agronomic and physiological growth characteristics, biomass production, and cannabidiol (CBD) and cannabigerol (CBG) yield were evaluated. Cannabinoid analysis of the female inflorescence extracts was performed using liquid chromatography coupled with mass spectrometry (LC-MS).

**Results:**

The results showed that topping caused a significant reduction in plant height and an increase in the length of secondary shoots, with a stronger effect observed in the cultivar ‘Santhica 70’. The diameter of the main shoot was not significantly affected by the topping treatment. Topping led to a significant increase in both fresh and dry biomass, with the highest values recorded in plants topped above the 4th and 5th nodes. Regarding physiological parameters, topping at the 4th node was associated with an increased photosynthetic rate, transpiration rate, and stomatal conductance. Regarding cannabinoid composition, an increase in CBD content was observed in ‘Enectaliana’ and in CBG content, in ‘Santhica 70’, indicating that topping may act as a morphogenetic and physiological stress factor that enhances the biosynthesis of secondary metabolites.

**Conclusions:**

Topping significantly altered plant architecture, suppressing height and promoting branching, which increased biomass, particularly at the 4th and 5th nodes. These morphological changes were accompanied by enhanced physiological performance and secondary metabolism, elevating CBD in ‘Enectaliana’ and CBG in ‘Santhica 70’. Therefore, topping at the 4th or 5th node is recommended to maximize yield and phytochemical quality.

**Trial registration:**

Not applicable.

**Supplementary Information:**

The online version contains supplementary material available at 10.1186/s42238-026-00410-2.

## Introduction

Industrial hemp (*Cannabis sativa* L.) is an herbaceous species of the family Cannabaceae, originating from Central Asia. It is among the oldest cultivated crops, with evidence of cultivation dating back to the Neolithic period (Farinon et al. 2020). In Greece, the earliest reference to hemp appears in Herodotus (ca. 450 B.C.), who described its euphoric properties and its use in the production of ropes and textiles (Papastylianou et al. 2018, Folina et al. 2020). In recent decades, global interest in hemp has increased rapidly due to legislative reforms and a growing recognition of its industrial and pharmaceutical potential apart from its psychotropic properties (Hussain et al. 2021, Chavalina et al. 2025). This renewed interest is further sustained by the crop’s sustainability, adaptability, and high productivity potential (Visković et al. 2023).

Secondary metabolites are plant compounds with limited distribution among taxa. Although not essential for primary metabolism, they play crucial roles in plant defense mechanisms, tolerance to abiotic stresses, and the regulation of ecological interactions (Adesina et al. 2020). Their biosynthesis and accumulation are highly sensitive to environmental factors, resulting in considerable variation in their concentration (Yeshi et al. 2022, Nicolas-Espinosa et al. 2023). Owing to its rich and diverse metabolic profile, hemp has emerged as a model species for the study of secondary metabolism, with over 750 metabolites identified to date – cannabinoids are the most characteristic (Small 2017, Mostafaei Dehnavi et al. 2022, Barčauskaitė et al. 2022).

Among cannabinoids, cannabidiol (CBD) is non-intoxicating and has well-documented therapeutic properties, i.e., anticonvulsant, anxiolytic, antipsychotic, anti-inflammatory, analgesic, neuroprotective, antioxidant, muscle relaxant, and immunomodulatory, and, thus, many applications in the pharmaceutical, food, and cosmetic industries (Pisanti et al. 2017, Arkell et al. 2019). Cannabigerol (CBG) – another non-intoxicating cannabinoid – not only displays a distinct spectrum of biological activities (anti-inflammatory, neuroprotective, analgesic, and potential anticancer effects) but also occupies a central position in the cannabinoid biosynthetic pathway. Specifically, the acidic form of CBG, cannabigerolic acid (CBGA), serves as the precursor molecule for the biosynthesis of cannabidiolic acid (CBDA) and Δ⁹-tetrahydrocannabinolic acid (THCA), and therefore CBD and Δ⁹-THC, the main psychoactive cannabinoid (Anokwuru et al. 2022, Calapai et al. 2022, Jastrząb et al. 2022). This precursor-product relationship indicates that an enhanced metabolic flux through CBGA pathways can influence the downstream biosynthesis of CBD. Consequently, the choice of cultivar or the implementation of agronomic practices that favor CBGA accumulation may modulate CBD yield and cannabinoid composition (Burgel et al. 2020, Park et al. 2022).

The genetic background of *Cannabis sativa* L. largely determines its cannabinoid profile, as different genotypes display distinct concentrations and ratios of those compounds (Bakel et al. 2011). However, environmental conditions and cultivation practices can also influence both cannabinoid biosynthesis and final accumulation, thereby modifying the composition and yield of cannabinoids (Gorelick and Bernstein 2017). Specifically, a range of cultivation conditions has been demonstrated to affect cannabinoid production, including light intensity (Rodriguez-Morrison et al. 2021), light quality (Danziger and Bernstein 2021), fertilization practices (Saloner et al. 2024), and nitrogen nutrition (Song et al. 2023).In addition to biochemical traits, physiological indicators such as chlorophyll content, photosynthetic rate, and stomatal conductance have been shown to reflect plant responses to stress and cultivation practices, providing insights into the mechanisms linking environmental stimuli to secondary metabolism (Caplan et al. 2017, Saloner and Bernstein 2020).

Controlled-environment experiments have also proven valuable in elucidating how cultivation conditions influence hemp morphology, physiology, and cannabinoid production (Amaducci and Gusovius 2010, Tang et al. 2016). Among agronomic practices, topping (the removal of the apical meristem) is a widely applied technique in various crops to optimize canopy architecture and yield. By removing apical dominance, this practice suppresses vertical elongation and redistributes hormonal signals, particularly auxins, toward lateral buds, thereby stimulating branching and altering the plant’s structural profile (Leonte et al. 2015, Bajić et al. 2022). These architectural changes improve intra-canopy light penetration and have been shown to positively affect photosynthetic performance. For instance, in cotton, chemical topping has been reported to significantly alter biomass partitioning and source–sink relationships, ultimately enhancing dry matter accumulation and yield components (Nie et al. 2021). Similarly, in tobacco, topping was found to increase net photosynthetic rate, modify nutrient absorption and redistribution, and improve overall photosynthetic efficiency, highlighting a direct link between architectural manipulation and physiological function (Shi et al. 2025). In *Cannabis sativa* L., recent studies have demonstrated that topping significantly increases plant dry weight and inflorescence yield (Danziger and Bernstein 2021, Danziger and Bernstein 2021, Roussis et al. 2022) and is often associated with elevated CBD concentrations, a finding of notable importance given the growing demand for CBD-rich products (Nachnani et al. 2021, Crispim Massuela et al. 2022).

Despite these promising results, comprehensive data on the physiological and biochemical effects of topping in hemp remain limited, particularly within the emerging Greek hemp sector where evidence-based agronomic guidelines are lacking. To address this gap, the present study introduces a holistic approach by integrating morphological, physiological, and biochemical evaluations to assess the response of two monoecious industrial hemp cultivars to varying topping intensities. The innovative aspect of this research lies in correlating specific architectural changes with photosynthetic efficiency and cannabinoid profile shifts, ultimately aiming to establish optimized, region-specific agronomic protocols.

## Materials and methods

### Plant Material, experimental Design, and growth conditions

A pot experiment was conducted under greenhouse conditions from April to June 2024 at the Department of Agriculture, University of Patras, Greece (38°21′59.2″ N, 21°28′35.5″ E).

**Fig. 1:**
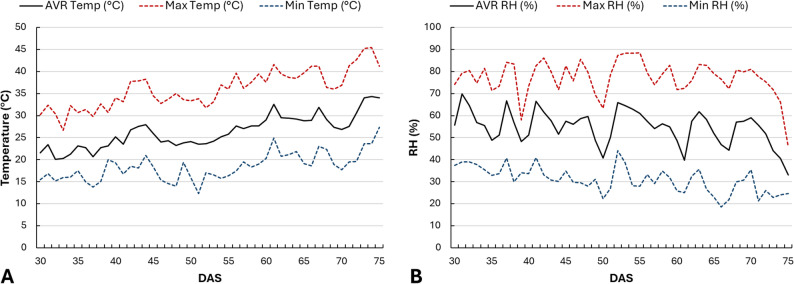
Daily variation of greenhouse temperature and relative humidity

During the experimental period, the average temperature was 26.0 °C, while relative humidity (RH) was 54.4% (Fig. 1). Air temperature and RH were recorded using a HOBO MX2301A Temperature/RH Data Logger (Onset Computer Corporation, Bourne, MA, USA) positioned at canopy height. Data were recorded at 1-hour intervals, and daily mean, maximum, and minimum values were computed from the hourly dataset. Plants were grown under natural photoperiodic conditions, which ranged from 13 to 15 h of daylight throughout the experiment.

The experimental layout followed a Completely Randomized Design (CRD) under greenhouse conditions with two factors: hemp cultivar and topping treatment. Two monoecious industrial hemp (*Cannabis sativa* L.) cultivars, ‘Enectaliana’ and ‘Santhica 70’, were used, and their main characteristics are summarized in Table 1. Seeds of ‘Enectaliana’ were supplied by Enecta S.r.l. (Italy), while seeds of ‘Santhica 70’ were supplied by Hempoil Greece. ‘Enectaliana’ is characterized by a relatively short cultivation cycle, while ‘Santhica 70’ requires a longer growth period to reach full maturity. The topping treatment was applied at four levels: no topping plants (Control), topping above the 4th node, topping above the 5th node, and topping above the 6th node from the base of the main stem. Topping was performed at 29 DAS, when plants were in the active vegetative growth stage, using a sterilized pruning shear. This timing was chosen to ensure that plants had developed sufficient nodes to allow for the specific topping levels while still maintaining vigorous vegetative growth. In total, eight treatment combinations (2 cultivars × 4 topping levels) were established, each consisting of seven replicates (one plant per pot), resulting in a total of 56 plants, randomly arranged within the greenhouse.

**Table 1:** Main characteristics of the two industrial hemp (*Cannabis sativa* L.) cultivars used in the experiment

Characteristic	‘Enectaliana’	‘Santhica 70’
Country of origin	Italy/Netherlands	France
Genotypic expression	Monoecious	Monoecious
Plant height at maturity	2.0–2.5 m	2.0–3.0 m
THC content	0.2–0.3%	≤ 0.2%

Seeds were sown in multipot trays filled with a commercial substrate (SUPER TERRA ST2, Hawita Professional), placing one seed per pot. The trays were placed in a controlled-environment growth chamber (Probox Basic, Garden High Pro) maintained at 25–26 °C, 55–60% RH, and a 16-h photoperiod. Seedlings were kept in the chamber for 10 days and subsequently transferred to the greenhouse for acclimatization to ambient conditions.

At 10 days after sowing (DAS), seedlings were transplanted into 10 L black plastic pots containing 3 kg of a homogeneous growth substrate prepared at a ratio of 2:2:1 (clay soil: peat: perlite, respectively). The soil component (non-sterilized) was collected from an experimental field adjacent to the greenhouse, whereas the commercial peat (Super Terra ST2, Hawita Professional, Germany) and perlite (Geoflor, Perlite Hellas S.A., Greece) used in the mixture were supplied as sterilized substrates. Technical characteristics of the growing substrate are presented in Table 2.

**Table 2:** Technical characteristics of the growing substrate used for the cultivation of industrial hemp plants

Technical characteristic	Description
Country of production	Latvia
Light peat, medium structure	0–10 mm
Black peat, fine structure	0–5 mm
Perlite content	10%
Electrical conductivity (EC)	< 1.2 mS/cm
pH (acidity)	5.5–6.0.5.0
Nitrogen (N)	140 mg/L
Phosphorus (P)	160 mg/L
Potassium (K)	180 mg/L
Volume	250 L

### Growth and yield parameters

Plant harvesting was carried out on 22 June 2024 (75 DAS) at the full flowering stage, when the trichomes of female inflorescences exhibited a color change from white to milky white. At harvest, key agronomic traits were evaluated, including plant height, stem diameter, length of secondary branches, and fresh and dry aboveground biomass.

From each treatment, five plants were selected and cut just above the substrate surface. Plant height was measured from the stem base to the apical meristem, while stem diameter was determined at the base of the main stem using a digital caliper. The length of secondary branches was measured from their point of emergence on the main stem to their apices using a measuring tape.

For dry weight determination, aboveground biomass samples were oven-dried in a forced-air circulation oven at 60 °C for 48 h until constant weight was reached. Measurements of fresh and dry biomass were conducted using a precision balance (KERN PFB, KERN & Sohn GmbH, Balingen, Germany; readability: 0.001 g).

Two plants per treatment were used for chemical analysis of secondary metabolites. Their inflorescences were manually removed, air-dried in a shaded and well-ventilated environment, and subsequently subjected to LC-MS analysis to determine the cannabidiol (CBD) and cannabigerol (CBG) contents.

### Physiological parameters

Total chlorophyll content (Chl, expressed as relative chlorophyll index units) was estimated using a portable chlorophyll meter (CCM-200, OPTI-SCIENCES, Hudson, NH, USA), following the manufacturer’s protocol. Measurements were taken on the two opposite, fully expanded leaves located at the 4th node of the main stem. From each leaf, three morphologically uniform leaflets (typically the central ones) were selected for evaluation. For every selected leaflet, five consecutive readings were recorded, resulting in a total of fifteen measurements per plant. Measurements were performed at four consecutive growth stages (30, 40, 50, and 60 DAS) to monitor the temporal progression of physiological parameters during plant development.

Gas-exchange parameters, including net photosynthetic rate (Pn, µmol m^− 2^ s^− 1^), transpiration rate (E, mmol m^− 2^ s^− 1^), and stomatal conductance (gs, mol m^− 2^ s^− 1^), were measured non-destructively using a portable photosynthesis system LCproSD (ADC BioScientific Ltd., Hoddesdon, Herts, UK). Measurements were conducted under controlled chamber conditions (flow rate: 200 mL min^− 1^; leaf temperature: 25 °C; ambient CO_2_ concentration automatically regulated). For the evaluation of physiological parameters, two opposite, fully expanded leaves at the 4th node from the base of the main stem were selected, ensuring direct exposure to natural sunlight. From each leaf, three successive measurements were recorded at 30-second intervals.All measurements were conducted during the morning hours (09:00–12:00 a.m.) to minimize the influence of high temperature and excessive transpiration, thereby ensuring physiological stability during data collection. Measurements were repeated at 30, 40, 50, and 60 DAS to monitor the dynamics of the plants’ physiological performance throughout the growth cycle.

### Extraction and LC-MS analysis

The extraction and analysis of cannabinoids was carried out following the protocol by (Ioannidis et al. 2025). Briefly, 1 g of dried, ground hemp inflorescences was precisely weighed and transferred to a round-bottom flask. Ultrasound-Assisted extraction was performed by adding 10 mL of methanol (Methanol 99.8% HPLC grade, Fisher Chemical), ensuring a hemp to solvent ratio of 1:10 (w/v) and incubating in an ultrasonic bath (ISOLAB Laborgeräte GmbH, Eschau, Germany) at 40 kHz and 120 W for 30 min, under reflux at ambient temperature (< 40 °C). The extract was filtered, and the residual plant material was returned to the extraction flask. The extraction procedure was repeated twice. The filtered extracts were combined and brought to dryness by removing the solvent using a rotary evaporator (RotaVapor R-205, Büchi Labortechnik AG, Flawil, Switzerland). The obtained dry extracts were weighed to determine the extraction yield, then they were re-dissolved in methanol at the appropriate concentration and filtered before analysis through a PTFE syringe filter (pore size: 0.20 μm, filter diameter: 15 mm, Chromafil, Macherey-Nagel, Düren, Germany).

High-performance liquid chromatography coupled to mass spectrometry (LC-MS) was used for the qualitative and quantitative analysis of the extracts. The instrumentation and chromatographic conditions used are described in a previous study (Ioannidis et al. 2025). For the chromatographic separation, a Kinetex^®^ Polar C-18 column (100 × 3.0 mm, 2.6 μm, 100 Å, Phenomenex) was used. The mobile phase consisted of 0.1% (v/v) formic acid and 10 mM of ammonium formate in H_2_O (mobile phase A), and 0.1% formic acid in MeOH (mobile phase B). A simple gradient elution was employed, starting from 67% to 87% B in 25 min, held at 87% for 3 min, then raised to 95% in 1 min and held at 95% for 5 min, decreased to 67% in 1 min and kept at 67% for 5 min for the re-equilibration of the column before the next injection (total run time: 40 min). The flow rate was 0.3 mL/min, the injection volume was 1 µL, and the column oven temperature was 30 °C.

### Statistical analysis

Experimental data were subjected to two-way analysis of variance (ANOVA) using IBM SPSS Statistics software, version 29.0.2.0 (IBM Corp., Armonk, NY, USA). Mean comparisons were performed using Tukey’s Honestly Significant Difference (HSD) test to evaluate statistically significant differences among the levels of the main factors and their interaction effects. All statistical tests were conducted at a 5% significance level (*p* < 0.05).

## Results

### Growth and yield parameters

**Fig. 2:**
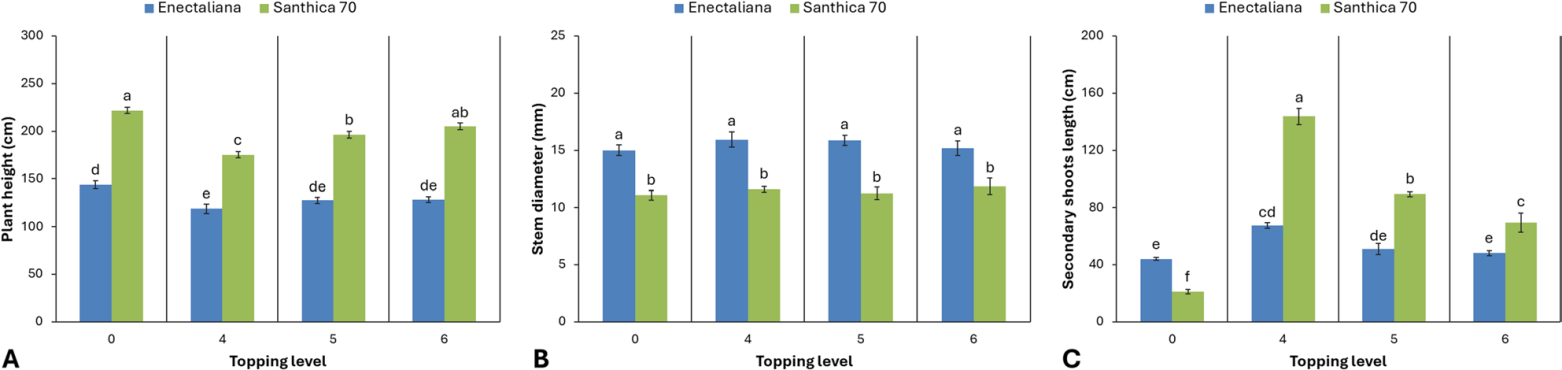
Morphological characteristics of hemp cultivars under different topping levels

**Fig. 3:**
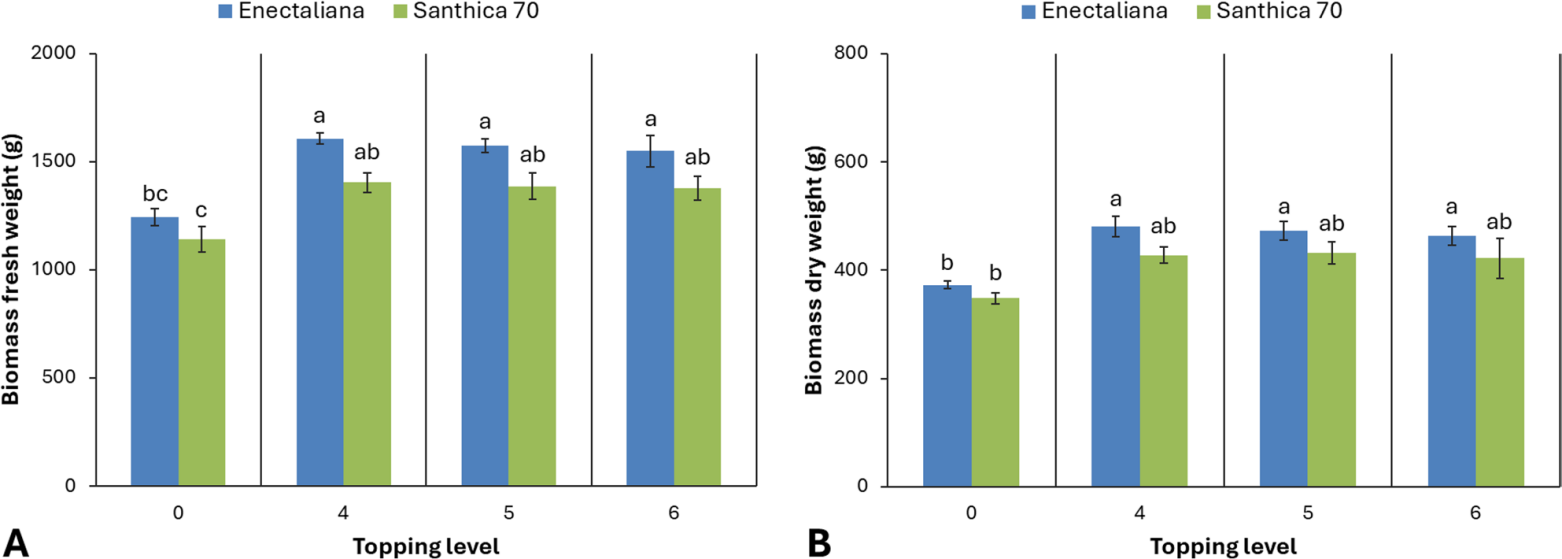
Biomass production of hemp cultivars under different topping levels

As shown in Figs. 2 and 3, the different levels of topping significantly affected all measured growth and biomass parameters, with distinct responses observed between the two cultivars, except for stem diameter, which remained unaffected by the treatment.

Plant height exhibited highly significant differences between cultivars and topping levels (*p* ≤ 0.001). The cultivar ‘Santhica 70’ showed consistently greater height than ‘Enectaliana’. In ‘Enectaliana’, non-topped plants (control) reached a mean height of 143.77 cm, whereas topping reduced height to 118.87, 127.70 and 128.70 cm after topping above the 4th, 5th, and 6th node, respectively. Similarly, in ‘Santhica 70’, the mean height of control plants was 221.87 cm, decreasing to 205.03 cm, 196.17 cm, and 175.10 cm after topping above the 6th, 5th, and 4th node, respectively.

Stem diameter differed significantly between cultivars (*p* ≤ 0.001) but was not influenced by topping (*p* > 0.05). ‘Enectaliana’ recorded greater mean values (14.98–15.92 mm) than ‘Santhica 70’ (11.08–11.85 mm).

The length of secondary shoots was significantly affected by both cultivar and topping (*p* ≤ 0.001). In ‘Enectaliana’, topping above the 4th node markedly increased shoot length to 67.50 cm compared with 44.10 cm in the control, while intermediate values were recorded for the 5th and 6th node treatments (50.90 and 48.10 cm, respectively). In ‘Santhica 70’, secondary shoot length ranged from 21.17 cm in control plants to 143.67 cm in plants topped above the 4th node, with significant increases also observed at the 5th (89.53 cm) and 6th node (69.43 cm).

Fresh biomass yield of the aerial parts differed significantly between cultivars and topping levels (*p* ≤ 0.001). In ‘Enectaliana’, the highest fresh weight was obtained from plants topped above the 4th node (1,606.48 g), followed closely by the 5th and 6th node treatments (1,573.55 and 1,549.02 g, respectively), while the control recorded a lower mean value (1,243.50 g). In ‘Santhica 70’, fresh biomass ranged from 1,141.59 g in the control to 1,402.89 g following topping above the 4th node.

A similar pattern was observed for dry biomass (*p* ≤ 0.001). ‘Enectaliana’ showed higher mean values (373.01–481.35 g) than ‘Santhica 70’ (348.41–431.48 g), with maximum dry biomass recorded in plants topped above the 4th or 5th node for both cultivars.

### Physiological Response of *C. sativa* L. to Topping: Effects on Total Chlorophyll Content and Photosynthetic Parameters

The two-way ANOVA revealed that both cultivar and topping significantly influenced physiological parameters (Chl, Pn, E, and gs), with effects varying according to developmental stage (30, 40, 50, and 60 days after sowing, DAS).

### Chlorophyll content (Chl)

No significant differences were observed in chlorophyll content at 30 DAS. However, the content was significantly affected by both cultivar and topping at 40 DAS (*p* ≤ 0.001). At later stages (50 and 60 DAS), topping continued to exert a significant influence (*p* ≤ 0.01 and *p* ≤ 0.05, respectively).

**Fig. 4:**
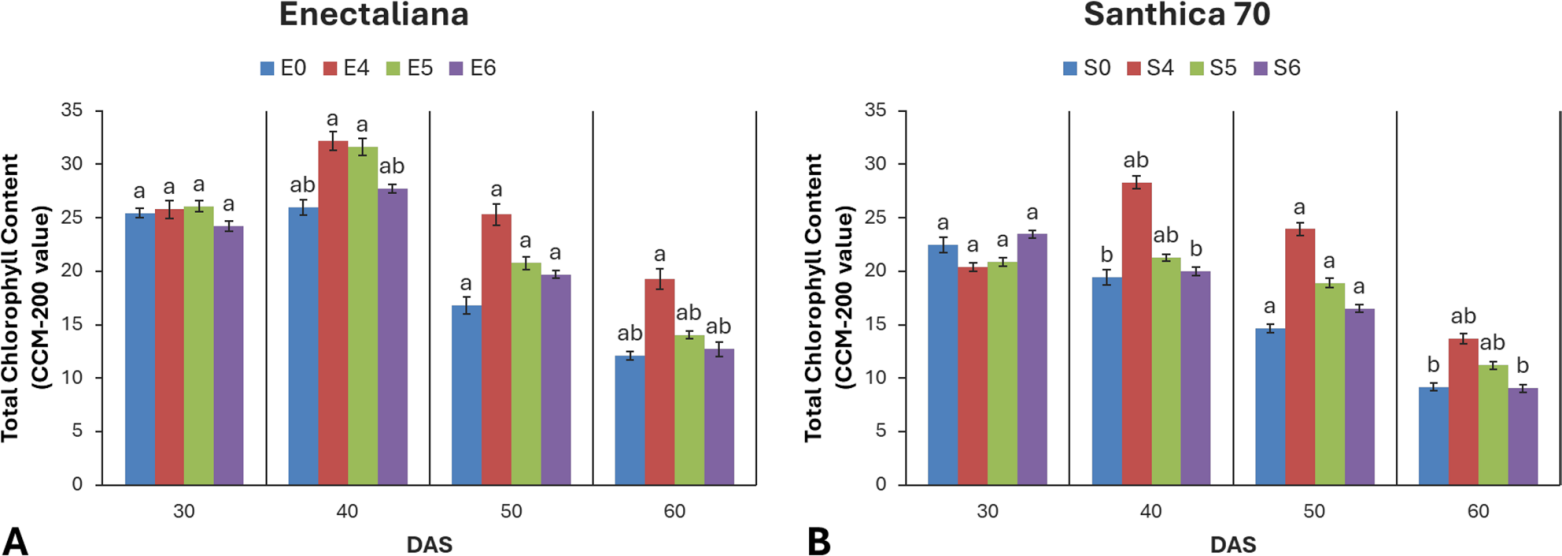
Total chlorophyll content of hemp cultivars under different topping levels

At 30 DAS, ‘Enectaliana’ exhibited 13.2% higher mean Chl values than ‘Santhica 70’ (25.43 vs. 22.47). At 40 DAS, differences became more pronounced. In ‘Enectaliana’, topping above the 4th node increased Chl by 24.1% relative to the control (32.21 vs. 25.96), while in ‘Santhica 70’ the increase reached 45.5% (28.32 vs. 19.46). At 50 DAS, topping maintained a positive effect (*p* ≤ 0.01), whereas cultivar differences were no longer significant. At 60 DAS, both factors again had significant effects (*p* ≤ 0.05), with ‘Enectaliana’ retaining higher mean Chl values (14.02) than ‘Santhica 70’ (11.27) (Fig. 4).

### Photosynthetic rate (Pn)

Significant effects of cultivar were observed at 30 and 40 DAS (*p* ≤ 0.05 and *p* ≤ 0.001, respectively), and strong effects of topping at 40, 50, and 60 DAS (*p* ≤ 0.001, *p* ≤ 0.001, and *p* ≤ 0.05, respectively). The cultivar × topping interaction was not significant at any stage (*p* > 0.05).

**Fig. 5:**
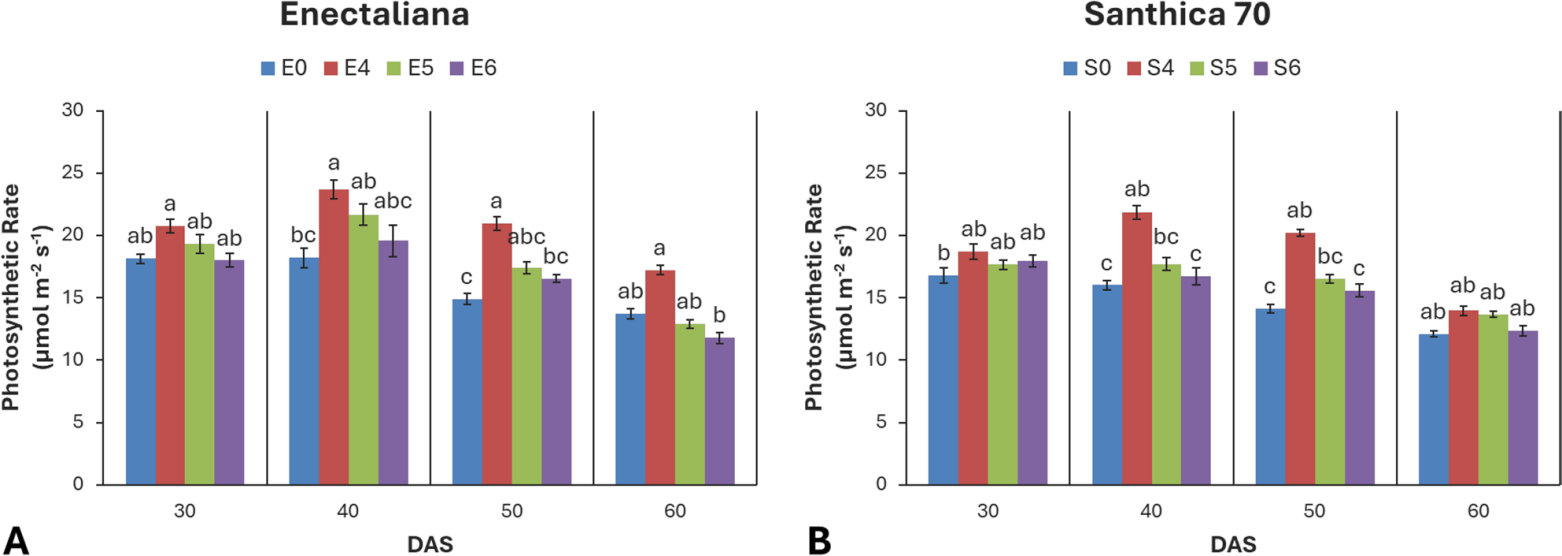
Photosynthetic rate of hemp cultivars under different topping levels

At 30 DAS, ‘Enectaliana’ showed a slightly higher photosynthetic rate (18.13 µmol m^− 2^ s^− 1^) than ‘Santhica 70’ (16.77 µmol m^− 2^ s^− 1^). Although topping had no significant effect at this stage, a mild increase (up to 14%) was recorded in plants topped above the 4th node. The strongest effect occurred at 40 DAS, when both cultivar and topping significantly enhanced Pn (*p* ≤ 0.001). In ‘Enectaliana’, the rate increased from 18.20 µmol m⁻² s⁻¹ in the control to 23.69 µmol m⁻² s⁻¹ in the 4th node treatment, while in ‘Santhica 70’ the increase reached 36.6% (16.01 to 21.88 µmol m^− 2^ s^− 1^). At 50 DAS, topping remained highly significant (*p* ≤ 0.001), with the 4th node treatment increasing photosynthesis by 40–45% relative to controls. At 60 DAS, photosynthetic rate continued to respond significantly to the topping treatment (*p* ≤ 0.05), while neither cultivar nor the cultivar × topping interaction showed significant effects. The 4th node topping consistently produced the highest Pn values in both cultivars, reaching 17.24 µmol m^− 2^ s^− 1^ in ‘Enectaliana’ and 13.97 µmol m^− 2^ s^− 1^ in ‘Santhica 70’ (Fig. 5).

### Transpiration rate (E)

No significant differences in transpiration rate were observed at 30 DAS. Significant cultivar effects were recorded at 40 DAS (*p* ≤ 0.001), while topping effects became significant at 40, 50, and 60 DAS (*p* ≤ 0.001, *p* ≤ 0.001, and *p* ≤ 0.05, respectively). No significant cultivar × topping interaction was detected at any stage (*p* > 0.05).

**Fig. 6:**
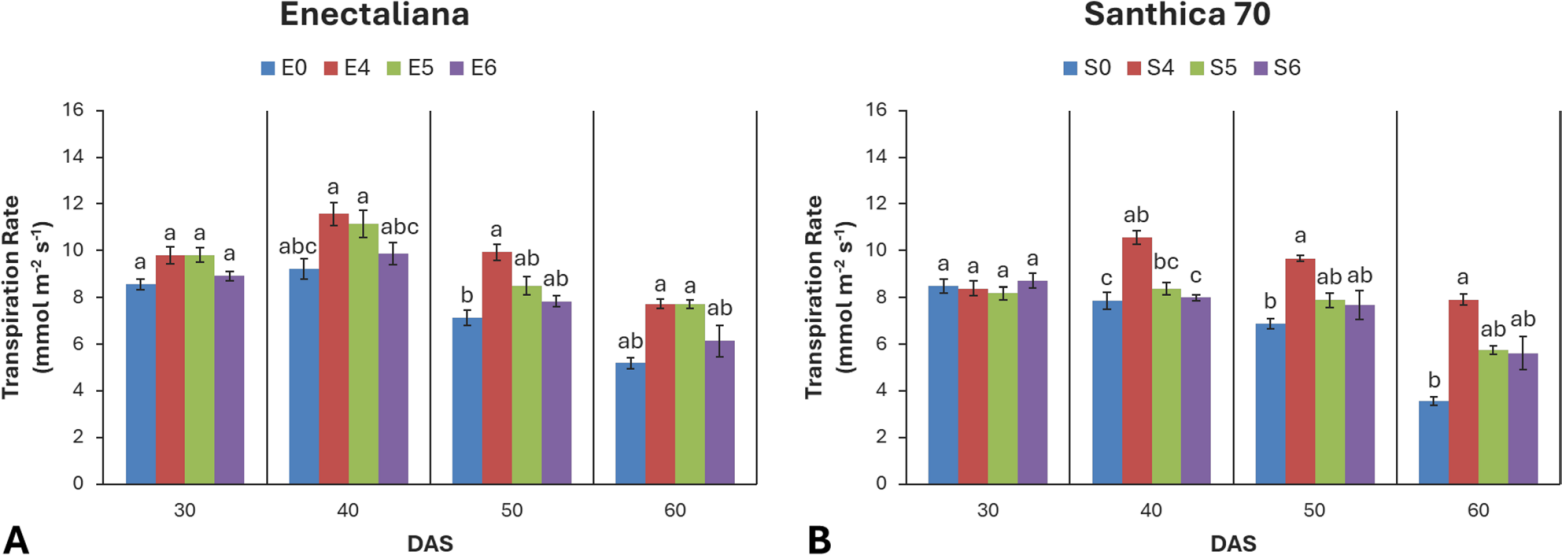
Transpiration rate of hemp cultivars under different topping levels

At 30 DAS, E ranged from 8.17 to 9.80 mmol m^− 2^ s^− 1^, with slightly higher values in ‘Enectaliana’ (9.80 mmol m^− 2^ s^− 1^). At 40 DAS, both cultivar and topping effects intensified (*p* ≤ 0.001), with ‘Enectaliana’ reaching a maximum of 11.57 mmol m^− 2^ s^− 1^ in the 4th node treatment, while ‘Santhica 70’ exhibited a 34.5% increase compared with the control. At 50 DAS, only topping remained highly significant (*p* ≤ 0.001), while cultivar differences were no longer detectable (*p* > 0.05). The highest E values were again observed in the 4th node treatments of both cultivars (9.93 mmol m^− 2^ s^− 1^ for ‘Enectaliana’ and 9.66 mmol m^− 2^ s^− 1^ for ‘Santhica 70’). At 60 DAS, only the topping (*p* ≤ 0.05) effect was significant (Fig. 6).

### Stomatal conductance (gs)

Stomatal conductance was mainly affected by topping at 40 and 50 DAS (*p* ≤ 0.001) and by cultivar at 40 DAS (*p* ≤ 0.05). No significant cultivar × topping interaction was detected.

**Fig. 7:**
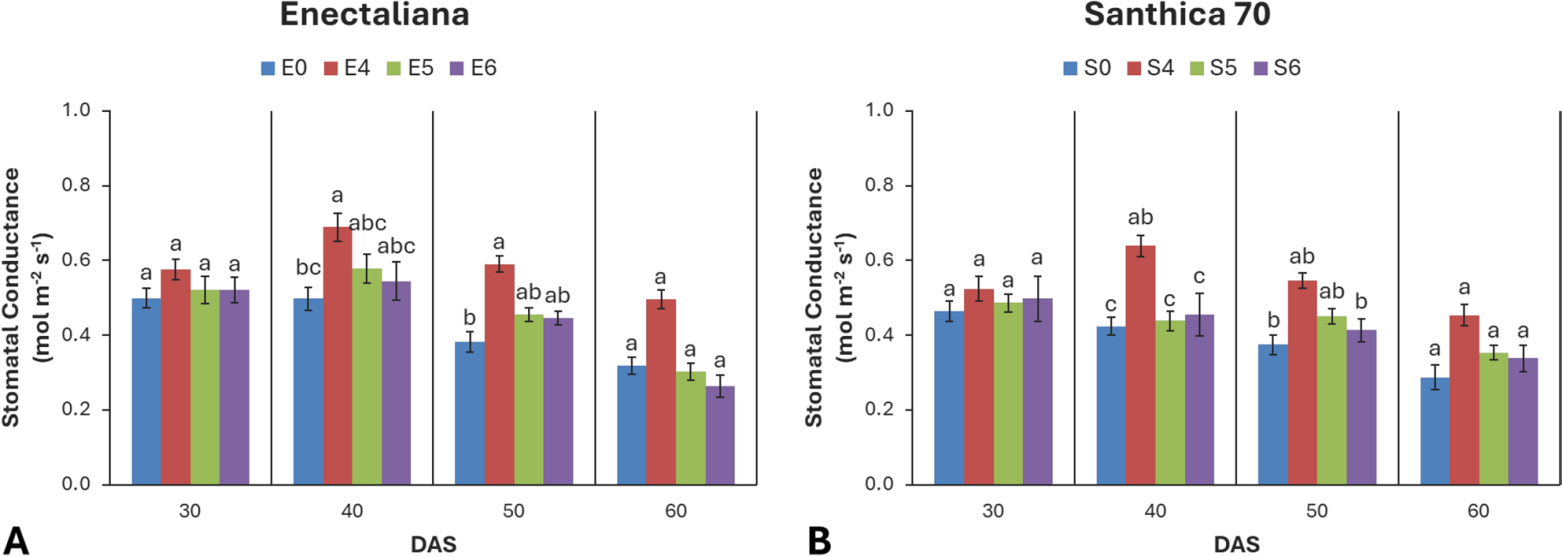
Stomatal conductance of hemp cultivars under different topping levels

At 30 DAS, gs ranged from 0.46 to 0.58 mol m^− 2^ s^− 1^, with ‘Enectaliana’ showing slightly higher values. At 40 DAS, both factors were significant (*p* ≤ 0.01 and *p* ≤ 0.001, respectively). ‘Enectaliana’ reached 0.69 mol m^− 2^ s^− 1^ at the 4th node treatment, while ‘Santhica 70’ reached 0.64 mol m^− 2^ s^− 1^. At 50 DAS, topping remained highly significant, peaking at 0.59 and 0.55 mol m^− 2^ s^− 1^ for ‘Enectaliana’ and ‘Santhica 70’, respectively. At 60 DAS, differences were no longer significant, though a general decline in gs (0.26–0.50 mol m^− 2^ s^− 1^) was observed across all treatments (Fig. 7).

### Cannabinoid content

LC-MS analysis of the main cannabinoids revealed that ‘Enectaliana’ predominantly accumulates CBD and its acidic precursor CBDA, whereas ‘Santhica 70’ is characterized by higher levels of CBG and CBGA, consistent with previous reports (Mazzara et al. 2022, Tagliatti Albreht 2021). In both cultivars, the acidic forms were more abundant than their neutral counterparts across all treatments.

**Fig. 8:**
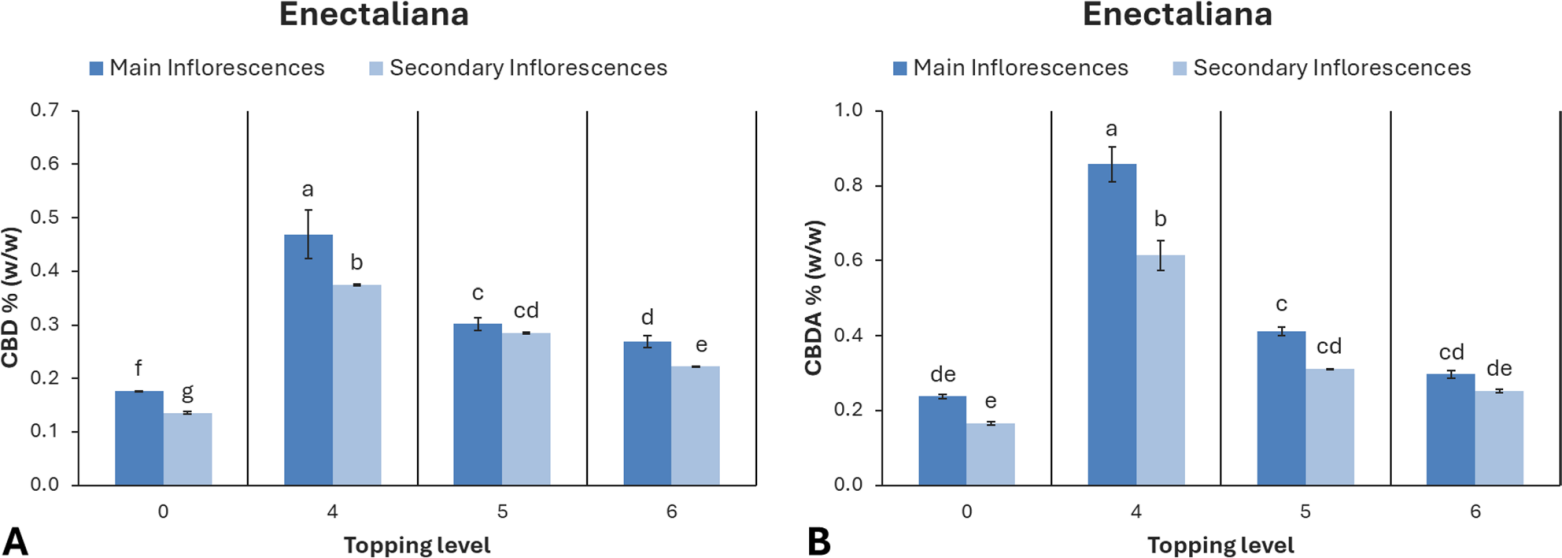
Cannabinoid content of hemp cultivar ‘Enectaliana’ under different topping levels

The concentrations of CBD and CBDA were significantly influenced by both inflorescence position (main or secondary) and topping treatment in ‘Enectaliana’ (Fig. 8). In the main inflorescences, the control exhibited the lowest mean CBD concentration (0.18%), while topping above the 4th node produced a significantly higher mean value (0.47%), surpassing all other treatments. In secondary inflorescences, CBD ranged from 0.14% in controls to 0.37% in the 4th node treatment, with intermediate values recorded for the 5th and 6th nodes (0.28% and 0.22%, respectively). The highest CBDA concentration was also observed in plants topped above the 4th node, reaching 0.86% (main inflorescences) and 0.61% (secondary inflorescences). Treatments at the 5th and 6th node yielded lower values, and the control recorded the lowest concentrations (0.24% and 0.17%, respectively).

**Fig. 9:**
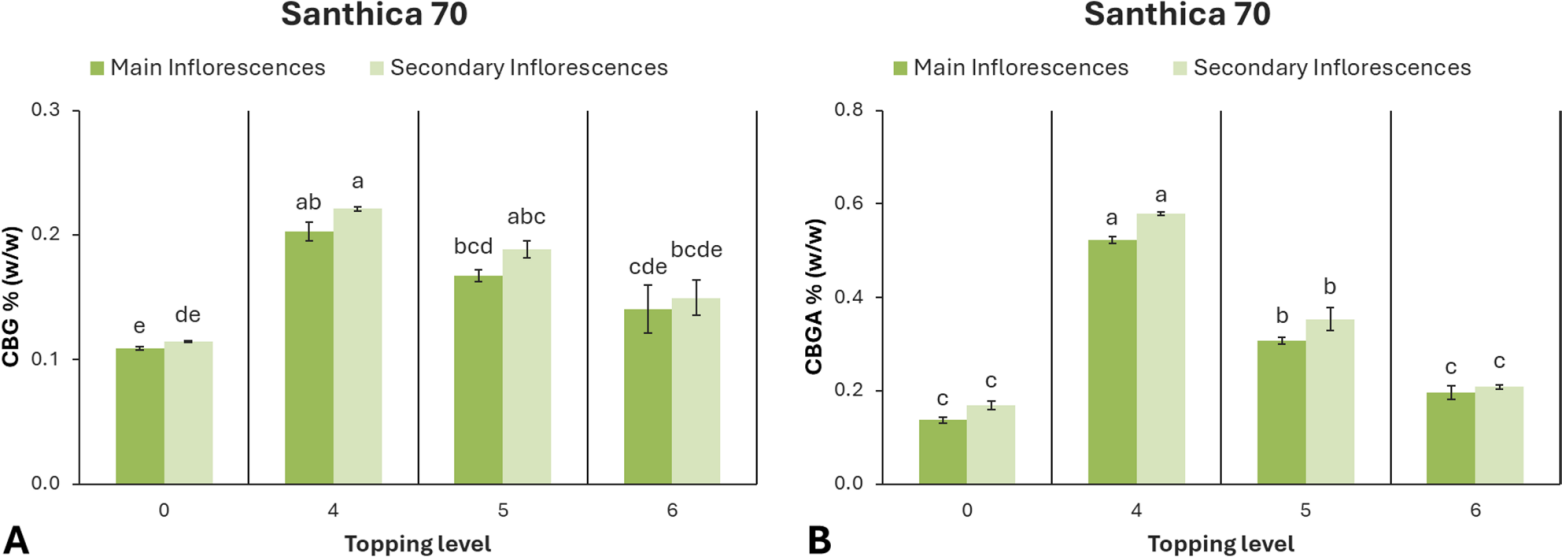
Cannabinoid content of hemp cultivar ‘Santhica 70’ under different topping levels

In ‘Santhica 70’, the concentrations of CBG and CBGA were significantly affected by the level of topping (*p* < 0.001). The highest CBG levels were obtained at the 4th node treatment, both in main (0.20%) and secondary inflorescences (0.22%). Plants topped above the 5th and 6th nodes showed intermediate concentrations, while the control exhibited the lowest mean values. A similar trend was observed for CBGA, with the 4th node treatment producing the highest accumulation, followed by the 5th node treatment (Fig. 9).

## Discussion

This study investigated the effect of topping on two industrial hemp cultivars (‘Enectaliana’ and ‘Santhica 70’) grown under controlled greenhouse conditions. Despite considerable progress in understanding the influence of cultivation techniques on *Cannabis sativa* L., available data remain limited, particularly regarding the morphological, physiological, and biochemical responses to topping. The present study provides comprehensive insights into these responses, demonstrating that removing the apical meristem acts as a potent agronomic tool to modify plant architecture and boost secondary metabolite accumulation.

### Morphological responses to topping

The morphological differences observed between the two cultivars can be attributed to inherent genetic factors influencing growth patterns and resource allocation. ‘Santhica 70’, a fiber-type industrial hemp variety, maintained greater plant height across treatments, likely reflecting higher auxin and gibberellin activity promoting internodal elongation (Teichmann and Muhr 2015). In contrast, ‘Enectaliana’ exhibited a shorter, more compact canopy with pronounced lateral branching-traits linked to enhanced cytokinin activity and a shift in metabolic resources toward lateral organs. Similar varietal contrasts have been documented by (Burgel et al. 2020, Meijer and Keizer 1996, Spitzer-Rimon et al. 2022), emphasizing the genetic regulation of hormonal balance and meristem responsiveness.

Topping significantly altered plant morphology by reducing apical dominance and stimulating lateral shoot development. The strongest effect occurred when plants were topped at the 4th node, leading to a 17–25% height reduction relative to controls. This aligns with classical models of apical dominance, whereby removal of the terminal meristem reduces auxin levels, increases cytokinin accumulation in lateral buds, and induces branching (Shimizu-Sato and Mori 2001, Gaudreau et al. 2020). These findings are consistent with those of Kocjan Ačko et al. (2019), who reported that apical bud removal in hemp enhanced branching and overall yield potential. The resulting increase in lateral shoot length expands the photosynthetically active leaf area and improves light interception, which together enhance overall canopy photosynthetic capacity.

Stem diameter, a structural indicator of mechanical stability and vascular efficiency, differed significantly between cultivars but was not affected by topping. ‘Enectaliana’ exhibited thicker stems on average, suggesting enhanced cambial activity and assimilate allocation to structural tissues. Similar relationships between cambial development and mechanical strength have been observed in other fast-growing annuals (Ding et al. 2024).

Both cultivar and topping strongly influenced biomass accumulation. ‘Enectaliana’ consistently produced higher fresh and dry biomass. The 4th node topping treatment maximized biomass production relative to controls. These results corroborate previous findings that topping enhances vegetative growth and aboveground yield (Folina et al. 2020, Roussis et al. 2022, Caplan et al. 2018), confirming its utility in optimizing biomass output in controlled environments.

### Physiological responses to topping

Total chlorophyll content (Chl) and photosynthetic parameters (Pn, E, gs) are reliable indicators of plant physiological performance and adaptive capacity under cultivation practices. In this study, both cultivar and topping significantly affected photosynthetic function, with the response magnitude depending on the developmental stage. ‘Enectaliana’ exhibited consistently higher chlorophyll content at all stages, indicating superior photosynthetic efficiency compared with ‘Santhica 70’. The most pronounced effects were recorded at 40 DAS, when topping at the fourth node increased Chl by 25–45% relative to controls. Although a gradual decline occurred later (50–60 DAS) due to natural senescence, topped plants maintained substantially higher values, suggesting delayed leaf aging and sustained photosynthetic activity.

Similarly, topping enhanced photosynthetic rate, transpiration, and stomatal conductance. At 40 DAS, topped plants of both cultivars displayed superior net photosynthetic rates (Pn), accompanied by higher E and gs, reflecting more efficient CO_2_ uptake and gas exchange without excessive water loss. Toward the end of the growth cycle (60 DAS), all parameters declined as plants entered reproductive maturation, yet topped plants retained higher values than controls.

The enhancement of photosynthetic activity following topping likely arises from both hormonal and structural adjustments. Apical removal disrupts auxin flow, elevates cytokinin concentration in upper tissues, and delays chlorophyll degradation, thereby sustaining high photosynthetic performance (Gaudreau et al. 2020, Madhumala et al. 2024). Moreover, canopy restructuring improves light penetration and air circulation, optimizing stomatal function, light use efficiency and internal CO_2_ conductance (Ciganda et al. 2009, Nkansah et al. 2021, Gu et al. 2023).

### Effects on secondary metabolism (CBD and CBG)

The variability of *C. sativa* cannabinoid composition is influenced by both cultivation practices and genetic background among cultivars (Mazzara et al. 2022, Tagliatti Albreht 2021). In this study, topping at the fourth node resulted in the highest cannabinoid content increase, both in the main and secondary inflorescences of ‘Enectaliana’ and ‘Santhica 70’ varieties, while higher topping positions led to less pronounced increases.

Appropriate canopy manipulation can stimulate secondary metabolism, especially cannabinoids, by triggering the plant’s defense mechanisms. Pruning changes plant’s architecture and improves light distribution, alters hormonal balance and promotes stress-related signaling, shifting resource allocation to increase precursor flux through the cannabinoid’s biosynthetic pathway. Even though topping does not alter the genetic profile of the plant, it improves its efficiency regarding the biosynthesis of secondary metabolites, including cannabinoids. As a result, total cannabinoid concentration increases, rendering the plants more potent.

These results underscore the importance of developmental timing and nodal position in modulating cannabinoid biosynthesis, in agreement with a previous study (Bernstein et al. 2019). Similar enhancements in CBD concentration following pruning or apical removal were reported by (Crispim Massuela et al. 2022) and (Dilena et al. 2023), reinforcing the potential of such practices for improving phytochemical yield.

### Practical implications and potential use as mother plants

Topping at the fourth node provided the optimal balance between height control, lateral shoot proliferation, and improved physiological activity, making it particularly suitable for the production of mother plants used in vegetative propagation. Increased lateral shoot availability and uniformity facilitate the collection of multiple cuttings of consistent quality – an approach widely applied in commercial propagation systems (Gaudreau et al. 2020, Caplan et al. 2018). Moreover, the enhanced physiological vigor (higher Pn, E, and gs) of topped plants may contribute to superior rooting success and faster establishment of clones.

## Conclusions

The present study demonstrated that apical bud removal (topping) significantly reduced plant height while promoting lateral shoot elongation, with a more pronounced effect in the cultivar ‘Santhica 70’. Stem diameter remained statistically unaffected by the topping treatment, suggesting that stem thickening is predominantly genotype-dependent rather than a result of apical pruning. Topping also led to a substantial increase in both fresh and dry aboveground biomass, with the highest yields recorded at the fourth and fifth nodes.

Physiologically, topping at the fourth node enhanced the net photosynthetic rate, transpiration, and stomatal conductance, indicating improved stomatal performance and greater efficiency in light and CO_2_ utilization. Regarding secondary metabolism, the treatment increased cannabidiol (CBD) accumulation in ‘Enectaliana’ and cannabigerol (CBG) in ‘Santhica 70’. This suggests that topping may act as a morphogenetic and physiological stimulus, triggering the biosynthesis of secondary metabolites.

Despite these promising findings, certain limitations of the present study should be acknowledged. The experiment was conducted under controlled greenhouse conditions and focused on two monoecious industrial hemp cultivars, which may limit the direct extrapolation of the results to open-field environments or to other genetic backgrounds. In addition, cannabinoid profiling was restricted to CBD and CBG, while other cannabinoids and terpenes were not evaluated. Future research should therefore expand the evaluation of topping practices to a broader range of cultivars and cultivation systems, including field conditions, and investigate different topping timings and intensities throughout the growth cycle. Moreover, integrating a more comprehensive metabolomic analysis, along with molecular and hormonal profiling, would provide deeper insights into the physiological mechanisms underlying the observed responses and further support the optimization of industrial hemp cultivation strategies.

## Supplementary Information

Supplementary Material 1

Supplementary Material 2

Supplementary Material 3

Supplementary Material 4

Supplementary Material 5

Supplementary Material 6

Supplementary Material 7

Supplementary Material 8

Supplementary Material 9

## Data Availability

All data generated or analyzed during this study are included in this published article and its supplementary information files.

## Abbreviations

CBD: Cannabidiol
CBG: Cannabigerol
CBDA: Cannabidiolic acid
CBGA: Cannabigerolic acid
CO_2_: Carbon dioxide
DAS: Days after sowing
DAT: Days after topping
DW: Dry weight
EC: Electrical conductivity
gs: Stomatal conductance
LC-MS: Liquid chromatography–mass spectrometry
MeOH: Methanol
P: Net photosynthetic rate
PTFE: Polytetrafluoroethylene
RH: Relative humidity
THC: Δ^9^-Tetrahydrocannabinol
FW: Fresh weight
