# Supplementary material for: Morphological, physiological, and biochemical responses of two industrial hemp (Cannabis sativa L.) cultivars to different levels of topping
Source: J Cannabis Res. 2026 Mar 6;8:55. doi: 10.1186/s42238-026-00410-2 (PMC13101382; doi:10.1186/s42238-026-00410-2)
Supplement: Supplementary file 1 — Supplementary Material 1 [file 42238_2026_410_MOESM1_ESM.docx]

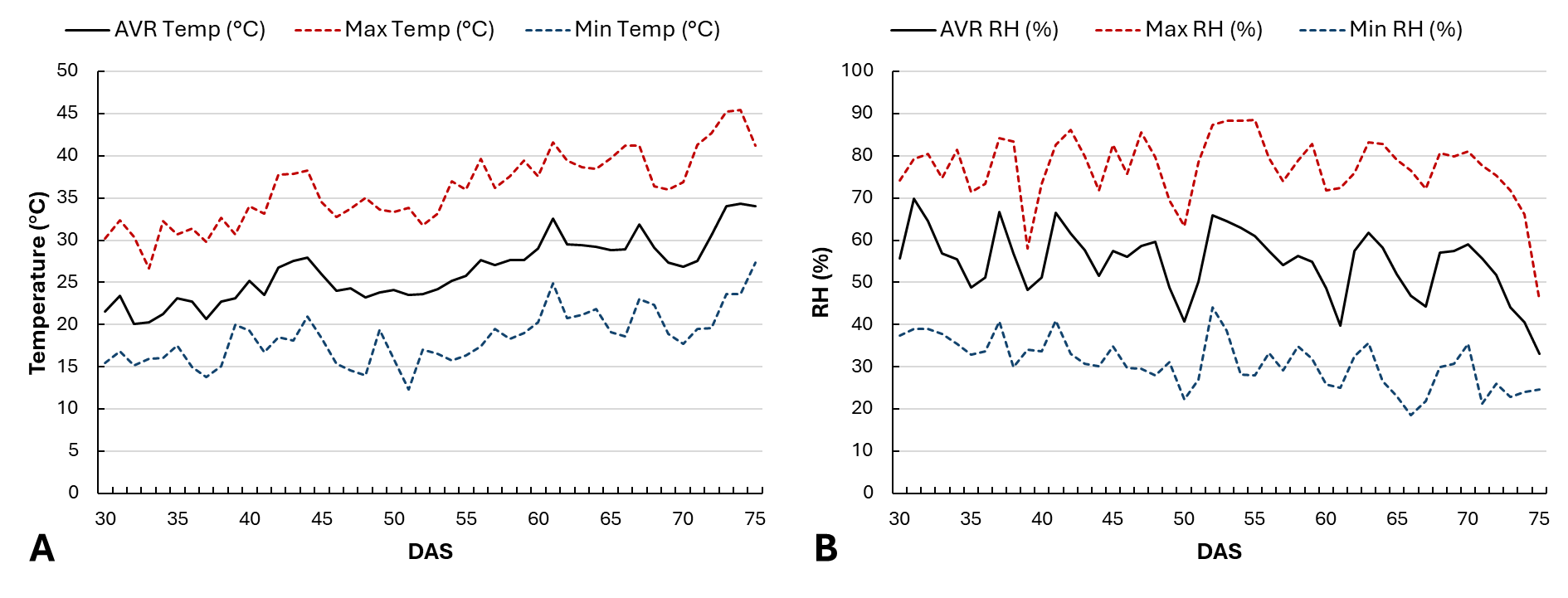


**Figure 1. Daily variation of greenhouse temperature and relative humidity.**

Daily variation of **(A)** temperature (°C) and **(B)** relative humidity (RH, %) recorded inside the greenhouse during the experimental period. The day corresponding to 30 days after sowing (30 DAS) represents the first day after the topping treatment. Data represent daily averages measured with a HOBO MX2301A Temperature/RH Data Logger (Onset Computer Corporation, USA).
