# Supplementary material for: Morphological, physiological, and biochemical responses of two industrial hemp (Cannabis sativa L.) cultivars to different levels of topping
Source: J Cannabis Res. 2026 Mar 6;8:55. doi: 10.1186/s42238-026-00410-2 (PMC13101382; doi:10.1186/s42238-026-00410-2)
Supplement: Supplementary file 4 — Supplementary Material 4 [file 42238_2026_410_MOESM4_ESM.docx]

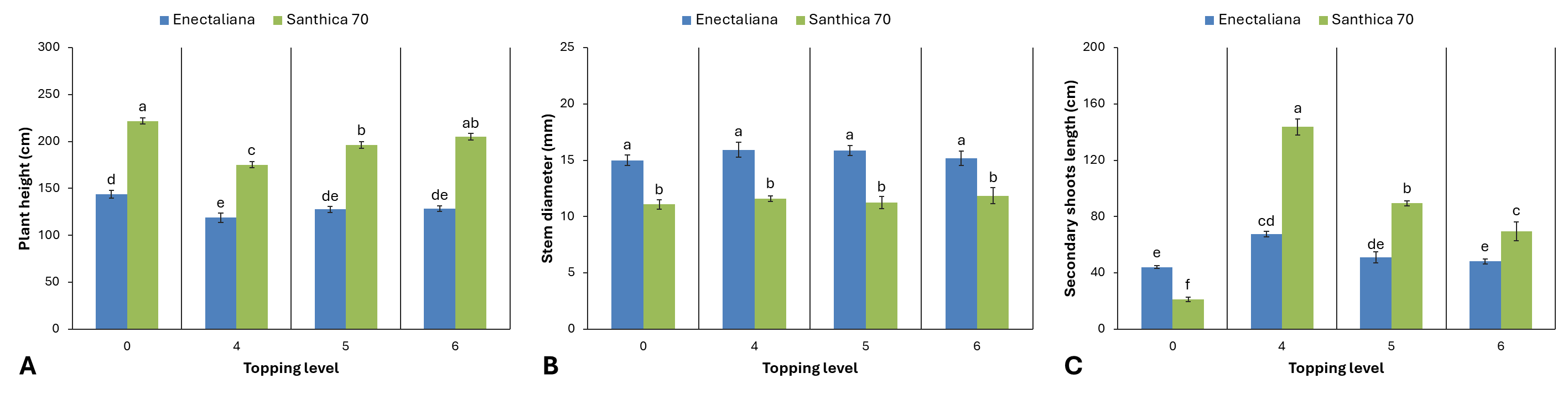


**Figure 2. Morphological characteristics of hemp cultivars under different topping levels.**

**(A)** Plant height, **(B)** stem diameter, and **(C)** secondary shoots length of *Cannabis sativa* L. cultivars ‘Enectaliana’ and ‘Santhica 70’ as affected by topping level (0 = control; 4 = topping above the 4th node; 5 = topping above the 5th node; 6 = topping above the 6th node). Data represent mean ± standard error. Different letters above bars indicate significant differences among treatments according to Tukey’s HSD test (*p* ≤ 0.05).
