# Supplementary material for: Morphological, physiological, and biochemical responses of two industrial hemp (Cannabis sativa L.) cultivars to different levels of topping
Source: J Cannabis Res. 2026 Mar 6;8:55. doi: 10.1186/s42238-026-00410-2 (PMC13101382; doi:10.1186/s42238-026-00410-2)
Supplement: Supplementary file 7 — Supplementary Material 7 [file 42238_2026_410_MOESM7_ESM.docx]

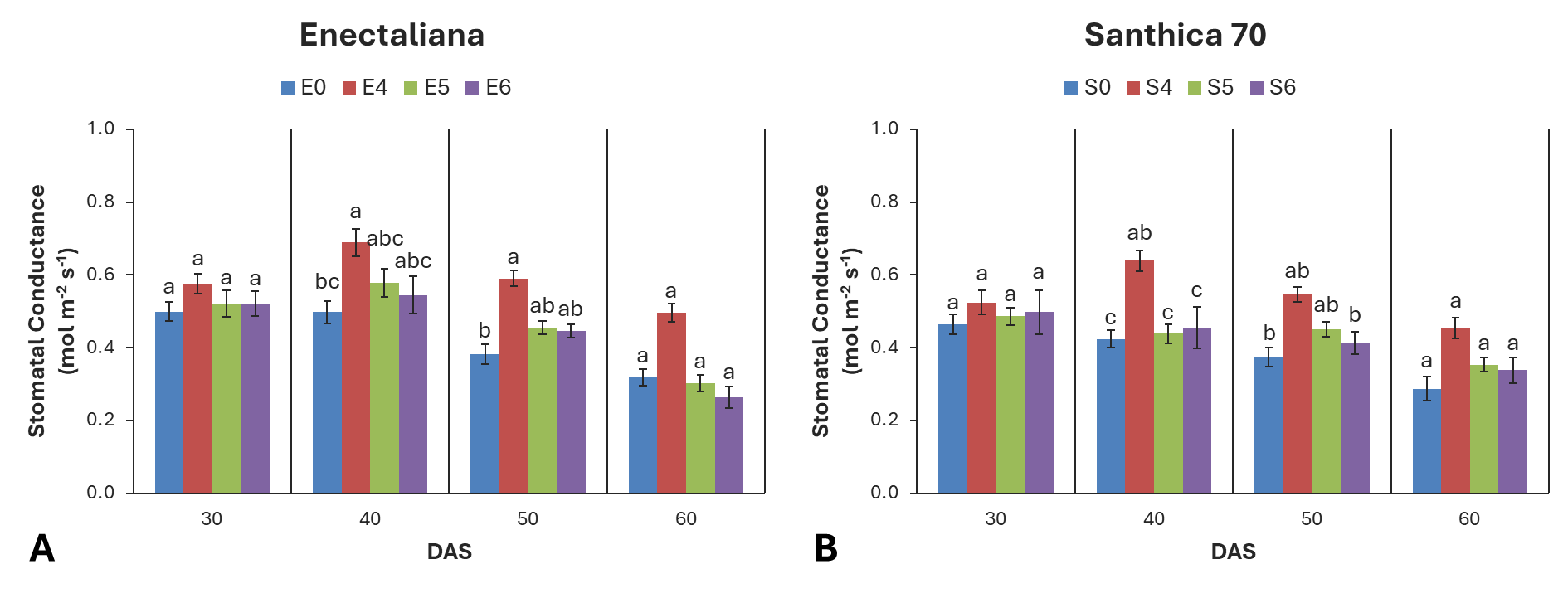


**Figure 7. Stomatal conductance of hemp cultivars under different topping levels.**

Stomatal conductance of *Cannabis sativa* L. cultivars **(A)** ‘Enectaliana’ and **(B)** ‘Santhica 70’ as affected by topping level (E0/S0 = control; E4/S4 = topping above the 4th node; E5/S5 = topping above the 5th node; E6/S6 = topping above the 6th node) at four growth stages (30, 40, 50, and 60 days after sowing, DAS). Data represent mean ± standard error. Different letters above bars indicate significant differences among all treatments (cultivars and topping levels combined) within each sampling date according to Tukey’s HSD test (*p* ≤ 0.05). The day corresponding to 30 days after sowing (30 DAS) represents the first day after the topping treatment.
